# Aspirin Has Antitumor Effects via Expression of Calpain Gene in Cervical Cancer Cells

**DOI:** 10.1155/2008/285374

**Published:** 2008-09-29

**Authors:** Sang Koo Lee, Min Seon Park, Myeong Jin Nam

**Affiliations:** ^1^Medical School, Hanyang University, Seoul 133-791, South Korea; ^2^Department of Research and Development, HanCell Inc, Incheon 406-799, South Korea; ^3^Department of Biomedical Science, National Institute of Health, Seoul 122-701, South Korea; ^4^Department of Biological Science, Gachon University of Medicine and Science, Incheon 406-799, South Korea

## Abstract

Aspirin and other nonsteroidal anti-inflammatory drugs show efficacy in the prevention of cancers. It is known that they can inhibit cyclooxygenases, and some studies have shown that they can induce apoptosis. Our objective in this study was to investigate the mechanism by which aspirin exerts its apoptosis effects in human cervical cancer HeLa cells. The effect of aspirin on the gene expression was studied by differential mRNA display RT-PCR. Among the isolated genes, mu-type calpain gene was upregulated by aspirin treatment. To examine whether calpain mediates the antitumor effects, HeLa cells were stably transfected with the mammalian expression vector pCR3.1 containing mu-type calpain cDNA (pCRCAL/HeLa), and tumor formations were measured in nude mice. When tumor burden was measured by day 49, HeLa cells and pCR/HeLa cells (vector control) produced tumors of 2126 mm^3^ and 1638 mm^3^, respectively, while pCRCAL/HeLa cells produced markedly smaller tumor of 434 mm^3^ in volume. The caspase-3 activity was markedly elevated in pCRCAL/HeLa cells. The increased activity levels of caspase-3 in pCRCAL/HeLa cells, in parallel with the decreased tumor formation, suggest a correlation between caspase-3 activity and calpain protein. Therefore, we conclude that aspirin-induced calpain mediates an antitumor effect via caspase-3 in cervical cancer cells.

## 1. Introduction

Aspirin and other agents characterized as nonsteroidal anti-inflammatory drugs (NSAIDs) are designed primarily to decrease pain and inflammation. The molecular basis for actions of NSAIDs is believed to be their ability to inhibit cyclooxygenase (COX) activity and block the production of prostaglandins [1]. Among NSAIDs, aspirin and sulindac can prevent the development of colon cancer and act as an anti-inflammatory agent by their inhibition of prostaglandin synthesis [2].

Cancer of the uterine cervix is the second leading cause of death from cancer in women worldwide and also the most prevalent gynecological malignancy in Korea [3]. We investigated whether aspirin induced apoptosis in human cervical cancer HeLa cells. To investigate the mechanism by which aspirin exerts its apoptosis effects, the effect of aspirin on the gene expression was studied by differential mRNA display RT-PCR (DD RT-PCR). Employing DD RT-PCR methods, we identified aspirin-responsive gene and mu-type calpain, which was confirmed by real-time quantitative PCR.

Calpain is known to possess the proteoglycanase activity in vitro [4]. Calpain is ubiquitous family of Ca^2+^-dependent neutral cysteine proteases. The two isoforms are classified according to their Ca^2+^ requirements: mu-type calpain and m-type calpain require micromolar and millimolar concentrations of Ca^2+^ for activation, respectively. Growing evidence suggested that calpain may play a central role in the execution of apoptosis via modulation of caspase-3 activity in glucocorticoid-treated and irradiated thymocytes, neuronal cells exposed to UV, or MCF-7 breast cancer cells treated with *β*-lapachone [5–7].

Further progress in cancer prevention would depend on understanding the mechanisms through which aspirin exerts molecular action. However, the molecular mechanisms through which aspirin alters colonic tumorigenesis are unknown. In this report, we describe a potential mechanism by which aspirin induces apoptosis in human cervical cancer cells. To examine whether mu-type calpain mediates antitumor effects in HeLa cells, HeLa cells were stably transfected with mu-type calpain cDNA. Tumor formation and caspase-3 activity of stably transfected cells were measured in nude mice. In this paper, we suggest that aspirin has an antitumor effect via the expression of mu-type calpain gene in cervical cancer cells.

## 2. Methods

### 2.1. Apoptosis Analysis

Human cervical cancer cells, HeLa, were plated in a 24-well plate at a density of 1 × 10^4^ cells/well and treated with various doses of aspirin. To detect an apoptotic body, cells were stained with Hoechst 33342 dye and assessed for morphological signs of apoptosis. The proportion of cells in G0/G1, S, and G2/M was determined by flow cytometric analysis of DNA content (Becton Dickinson, Peterson, NJ, USA). Cell suspension was stained with propidium iodide. DNA histograms were analyzed using CELL QUEST software to evaluate cell cycle compartments.

### 2.2. Differential mRNA Display RT-PCR and Cloning

Total RNA was extracted from cells with TRizol reagent (Invitrogen, Carlsbad, Calif, USA), following the protocol that was provided. Cell monolayers were washed with PBS, and 1 mL of TRizol/10^6^ cells with 4 units of RNase inhibitor were added. For each sample, 2 *μ*g of RNA were treated with DNase l (Roche, Basel, Switzerland) at 37°C for 30 minutes to remove contaminating DNA. One-base-anchored oligo-dT primers were used to reverse transcribed total RNA into first-strand cDNA, which were amplified subsequently by PCR using the arbitrary upstream primers. PCR products were labeled with 2 *μ*Ci of *α*[^32^P]dCTP (Amersham, Arlington, Ill, USA), and analyzed on a 6% polyacrylamide-urea gel. The cDNA bands that were unique to control or aspirin-treated cells were cut out of the gel, eluted, and reamplified by PCR. The candidate cDNA was cloned into pGEM-T vector (Promega, Madison, WI, USA). Plasmid with insert was purified and sequenced after performing PCR reactions.

### 2.3. Real-Time Quantitative PCR

We relied on the TaqMan assay (Perkin-Elmer model 7700; Foster City, CA, USA) to quantitate the amount of calpain mRNA. The forward and reverse primers and the FAM-tagged probe used for the mu-type calpain gene in the assay were 5′-GGATGTCATTCCGAGACT, 5′-CTCGTAGACCGCGAAG, and 5′-6FAM-TCTGCAACCTCACACCCGAC-TAMRA, respectively. The forward and reverse primers and FAM-tagged probe used for the ß-actin gene were 5′-AACTTGAGATGTATGAAGGCTTTTGG, 5′-TTTTTTTTTTTTTTTTTTTTTTTTTTTTTAAG, and 5′-6FAM-CAACTGGTCTCAAGTCAGTGTACAGGTAAGCCCT-TAMRA, respectively. To measure the relative abundance of the calpain gene in any given RNA sample, the amplification value derived using the calpain sequence was divided by the amplification value using the ß-actin sequence.

### 2.4. Transfection of Calpain cDNA and Cell Growth Assay

The mu-type calpain cDNA was retrieved with the following primers: forward 5′-AGGATGTCGGAGGAGA and reverse 5′-CCAGTACACAAGTCCCT. PCR reaction products were cloned into pCR3.1 vector (Invitrogen). The vector pCR or recombinant pCRCAL was stably transfected into HeLa cells by liposome. Control, vector-transfected (pCR/HeLa), and calpain-transfected cells (pCRCAL/HeLa) were counted by the trypan blue exclusion assay and Coulter counter (Coulter Corporation, FL, USA) for measuring stably transfected cell growth. Cell number was presented as the mean ± S.E. five experiments.

### 2.5. Enzymatic Assay for Caspase-3 Activity

Cells (1.5 × 10^6^) were plated in cell culture dishes (100 mm) and allowed to attach for 48 hours under cell culture conditions. Then the cells were treated and the activity of caspase-3 was measured using the fluorogenic enzyme substrates, z-DEVD-AFC (Molecular Probes, Eugene, Ore, USA). Samples were read in a fluorometer equipped with a 400 nm excitation filter and 505 nm emission filter. Enzyme activity was expressed as relative fluorescence units/mg of protein. The arbitrary values were presented as the mean ± S.E. five experiments.

### 2.6. Tumorigenicity

Balb/c nu/nu mice, 4–6 weeks of age, were acclimated and caged in groups of five. HeLa, pCR/HeLa, and pCRCAL/HeLa cells (1.5 × 10^7^) were injected subcutaneously into the right flank of the nude mouse. The mean tumor diameter was measured by dial caliper, and the volume was calculated by the formula: volume (mm^3^) = (square root of width × length)^3^. Mean values of five mice/group ±SEM are shown. The experiment was repeated three times and performed according to the guidelines of the Animal Experimental Committee, National Institute of Health, South Korea. Statistical calculations were performed using the Microsoft Excel 97 program (1998; Microsoft Co., Redmond, Wash, USA) to estimate *P*-value. The significance level (*P*-value) is determined using the Student's *t*-test. Probability values <.05 were considered significant.

## 3. Results

### 3.1. Apoptosis in Aspirin-Treated HeLa Cells

**Figure 1:**
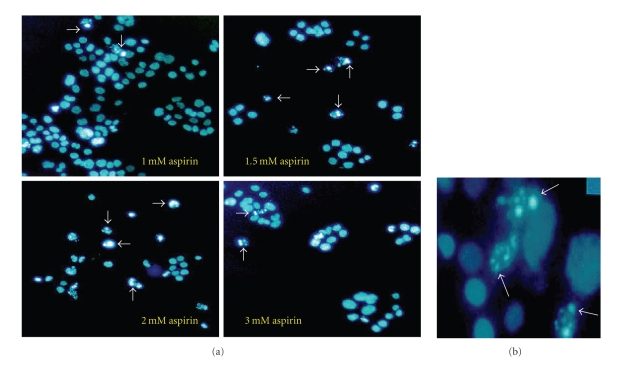
Morphological characteristics of HeLa cells after aspirin treatment. (a) Cells were treated with various concentrations of aspirin and grown for 48 hours. For detection of apoptotic morphology, cells were stained with Hoechst 33342 dye and assessed for morphological signs of apoptosis. The right panel (b) shows the enlarged photograph of apoptotic cells (×40).

We performed DNA synthesis assay to study the effects of aspirin on HeLa cells. HeLa cells were treated in 1, 2, or 3 mM aspirin. Aspirin inhibited growth of cervical cancer cells in a time- and concentration-dependent manner (data not shown). HeLa cells were then assessed for apoptosis. Aspirin-induced morphological changes were evident in a concentration-dependent manner (see Figure 1). Cells treated with aspirin became sparse, long squared, and detached from the dishes. Cell number was also decreased. Apoptotic bodies (indicated by white arrows in Figure 1) were shown after aspirin treatment. To show apoptosis in the cells treated with aspirin, flow cytometry analysis was performed. The population of sub-G1 phase was changed from 1.2 to 18.9% in cells treated with 1 mM aspirin for 48 hours. That of sub-G1 phase at 2 and 3 mM was in the similar range.

### 3.2. Identification of Calpain Gene in Aspirin-Treated HeLa Cells

**Figure 2:**
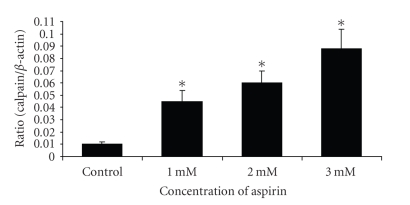
Real-time quantitative RT-PCR for calpain gene in aspirin-treated HeLa cells. To measure the relative abundance of mu-type calpain gene in a given RNA sample, the amplification value derived using calpain gene was divided by the amplification value using the *β*-actin sequence. ∗, *P* < .05 control versus treated group.

After HeLa cells were treated with aspirin for 48 hours, we performed differential display RT-PCR and selected differentially expressed genes, which was expressed with absolute difference between control and aspirin-treated cells. The genes were identified with DNA sequencing. One of the upregulated genes is mu-type calpain. Expression of calpain mRNA was confirmed by real-time quantitative PCR (see Figure 2). Calpain gene was highly expressed in aspirin-treated HeLa cells in a concentration-dependent manner. Calpain gene was upregulated by 4.4, 6, and 8.8 folds in the 1, 2, and 3 mM aspirin-treated HeLa cells, respectively.

### 3.3. Tumorigenicity of Calpain Gene Product

**Figure 3:**
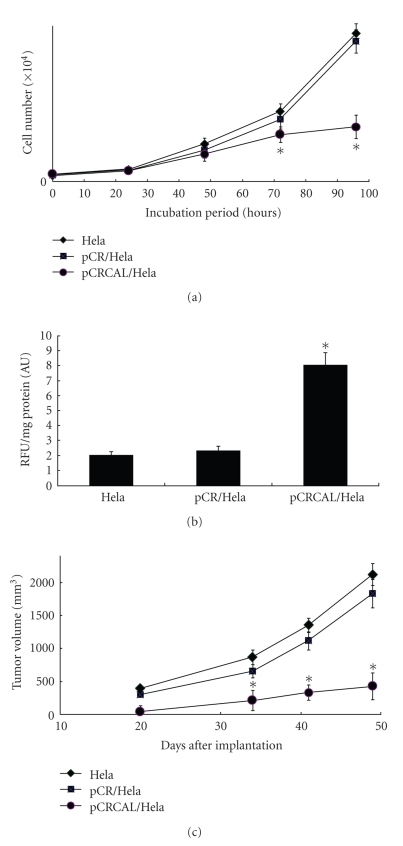
In vitro characteristics and tumorigenicity of stably transfected cells. (a) Cell proliferation. Control (HeLa), vector-transfected (pCR/HeLa), and mu-type calpain-transfected cells (pCRCAL/HeLa) were counted by the trypan blue staining and Coulter counter. (b) Caspase-3 activities. Caspase-3 activities were measured by analyzing the fluorometric cleavage of substrates, z-DEVD-AFC. RFU, relative fluorescence units. (c) Groups of five mice each were injected with HeLa, pCR/HeLa, and pCRCAL/HeLa cells (1.5 × 10^7^ in 0.1 mL saline) subcutaneously inducing solid tumor. The tumor volume was evaluated on the 20th, 34th, 41st, and 49th day onwards after tumor cell induction. The tumor volume was calculated by the formula: volume (mm^3^) = (square root of width × length)^3^. Mean values of five mice/group ±SEM are shown. The experiment was repeated three times with similar results. ∗, *P* < .05 HeLa versus pCRCAL/HeLa.

We have cloned mu-type calpain cDNA into pCR3.1 vector. The vector pCR or recombinant pCRCAL was stably transfected into HeLa cells (pCR/HeLa cell or pCRCAL/HeLa cell). To assess whether change of cell biology was caused after gene transfection, we measured cell proliferation. pCRCAL/HeLa cells appeared to have a markedly different growth pattern compared with HeLa cells and pCR/HeLa cells (see Figure 3(a)). To establish a relationship between calpain and caspase-3, caspase-3 activities were measured. As shown in Figure 3(b), caspase-3 activity was elevated in pCRCAL/HeLa cells. The markedly increased activity levels of caspase-3 in pCRCAL/HeLa cells suggest a correlation of caspase-3 activity and calpain protein. In investigation of calpain role in the tumorigenicity, we evaluated tumor progression in nude mice. 1.5 × 10^7^ stably transfected cells were subcutaneously injected into the flank of the mouse. When tumor burden was measured by day 49, HeLa cells and pCR/HeLa cells produced tumors of 2126 ± 163 mm^3^ and 1638 ± 213 mm^3^, respectively, while pCRCAL/HeLa cells produced markedly small tumor of 434 ± 206 mm^3^ in volume (see Figure 3(c)). The experiment was repeated three times with similar results. Tumor growth was reduced by calpain gene expression.

## 4. Discussion

We report that aspirin inhibited the proliferation of cervical adenocarcinoma cells in a time- and dose-dependent manner. This agrees with other studies showing that aspirin inhibited the proliferation of cancer cells [2, 4, 8–12]. Aspirin has antitumor effects in the colon through induction of quiescence and apoptosis [13]. Apoptosis was shown to be responsible for the cell growth inhibitory effects of aspirin in HT29 human colon carcinoma cells. Our morphological observation of nuclear condensation after aspirin treatment suggests that aspirin increases apoptosis in cervical cancer cells (see Figure 1). These results demonstrate that aspirin is an effective antitumor drug that induces apoptosis in cervical cancer cells.

A few molecular mechanisms of aspirin have been proposed. One of these mechanisms is COX-independent [14, 15]. It is well known that activation of p53 expression is involved [16]. In this study, we have shown that aspirin induces the calpain gene and calpain gene activation inhibits the tumor formation. These results support the previous findings that aspirin induces apoptosis by the regulation of bcl-2 and caspase-3 in human cervical cancer cells [3, 17]. Therefore, aspirin might play roles in the inhibition of tumor formation through activation of calpain gene.

Calpain is a calcium-dependent cysteine protease that is implicated in calcium-dependent cell death [17, 18]. Calpain plays an essential role in apoptotic commitment by cleaving Bax and generating the Bax/p18 fragment, which in turn mediates cytochrome c release and initiates the apoptotic execution [19]. Calpain activation plays a critical role in cancer cell adhesion and motility [20]; and calpain could be related to a therapeutic strategy targeting multiple disease states [21–24]. In our experiments, caspase-3 activity was increased in the calpain-transfected HeLa cells, suggesting a correlation of calpain and caspase-3 (see Figure 3(b)). The involvement of caspase-3 in the calpain action is in agreement with the results in the photoreceptor cell, where calpain executes apoptosis via modulation of caspase-3 activity [25]. Accompanying the increased caspase-3 activity, tumor growth was reduced by calpain gene expression, leading to the role of calpain as an apoptosis mediator (see Figure 3(c)). Fushimi et al. have analyzed calpain release from cultured chondrocytes stimulated by a proinflammatory cytokine, tumor necrosis factor-*α* (TNF-*α*) [4]. The effects of NSAIDs on calpain release were also examined. However, their results were in contrast to our expectation. NSAIDs examined (aspirin, loxoprofen-SRS, diclofenac sodium, indomethacin, and NS398) potently inhibited TNF-*α*-induced release of calpain [4]. In addition, they showed that PGE_2_ alone failed to stimulate, but it significantly augmented the release of calpain in the presence of 1 ng/mL TNF-*α* in HCS-2/8 cells. Moreover, inhibition of calpain release by an NSAID, loxoprofen-SRS, was significantly reversed by 100 nM PGE_2_. Therefore, further studies are necessary to clarify the relationship implicated in the aspirin treatment.

## 5. Conclusion

Aspirin causes growth inhibition of cervical cancer cells through activation of apoptosis. We suggest that aspirin may have cancer-preventing effects through calpain gene expression, which leads to caspase-3 activation.
